# Genome Sequence of Carbon Dioxide-Sequestering *Serratia* sp. Strain ISTD04 Isolated from Marble Mining Rocks

**DOI:** 10.1128/genomeA.01141-16

**Published:** 2016-10-20

**Authors:** Manish Kumar, Rajesh Kumar Gazara, Sandhya Verma, Madan Kumar, Praveen Kumar Verma, Indu Shekhar Thakur

**Affiliations:** a School of Environmental Sciences, Jawaharlal Nehru University, New Delhi, India; b National Institute of Plant Genome Research, New Delhi, India

## Abstract

The *Serratia* sp. strain ISTD04 has been identified as a carbon dioxide (CO_2_)-sequestering bacterium isolated from marble mining rocks in the Umra area, Rajasthan, India. This strain grows chemolithotrophically on media that contain sodium bicarbonate (NaHCO_3_) as the sole carbon source. Here, we report the genome sequence of 5.07 Mb *Serratia* sp. ISTD04.

## GENOME ANNOUNCEMENT

Increase in CO_2_ concentration can be mitigated by autotrophic and heterotrophic carbon fixation by plants and microorganisms (1). Some microorganisms, in addition to carbon dioxide fixation, also produce value-added products (2). *Serratia* sp. strain ISTD04 was isolated from marble mining rocks of the palaeoproterozoic metasediments of the Aravali Supergroup, Rajasthan, India, by enrichment in minimal salt media with increasing concentration of NaHCO_3_ as the carbon source (3). The bacterium is characterized by chemolithotrophic fixation of carbon dioxide which is supported by the presence of ribulose-1,5-bisphosphate carboxylase/oxygenase (RuBisCo), carbonic anhydrase, and carboxylases (4). The strain was studied in detail for CO_2_ sequestration along with biodiesel and polyhydroxyalkanoate (PHA) production (5, 6).

Whole-genome shotgun sequencing of *Serratia* sp. strain ISTD04 was performed on the Illumina Miseq platform and resulted in 2,073,386 paired-end reads of 151-bp length. After filtering the raw reads using NGS tool kit (v2.3.1), high-quality 1,485,793 paired-end reads were obtained. The genome assembly was performed using Velvet (v1.2.10) (7), SOAPdenovo (8), and gsassembler using a k-mer value of 79 for primary assembly. SSPACE (9) was used to perform scaffolding of the primary assembled contigs that generated 120 scaffolds, with an *N*_50_ scaffold size of 1,03,262 bp. The maximum scaffold length was 3,28,633 bp and minimum scaffold length was 210 bp. The NCBI prokaryotic genome annotation pipeline (PGAP) was used for the identification of candidate gene models. Furthermore, Pfam (10) annotation was carried out to assign functional domains to the predicted gene models. The pathways and the genes involved were predicted with the help of the KEGG Automatic Annotation Server (KAAS) (11).

The total genome size of *Serratia* sp. strain ISTD04 is 5.07 Mb with a G+C content of 59.98%. The assembly resulted in a coverage of 81×. A total of 4,563 protein coding gene models were predicted by PGAP. Moreover, 75 tRNAs, 8 rRNAS (2 5S rRNAs, 4 16S rRNAs, and 2 of 23S rRNAs), and 12 noncoding RNAs (ncRNAs) were also identified. In addition, 88 pseudo genes were predicted, among which were 6 frame-shifted pseudogenes. PGAP annotated 3,765 (82.51%) of the total predicted genes. In addition, Pfam annotation was assigned to 4,234 genes (92.78%) and 1,498 genes (32.82%) were predicted by the KAAS tool to be involved in various pathways.

*Serratia* sp. ISTD04 is a novel organism which performs chemolithoautotrophic carbon dioxide assimilation. Genome analysis of *Serratia* sp. ISTD04 revealed the presence of phosphoribulokinase (PRK) and other CBB pathway genes. However, the RuBisCo gene could not be identified in the genome assembly (12). The carbonic anhydrase, an important facilitator enzyme, is also present in the genome (13). Anaplerotic CO_2_ assimilating enzymes *viz*. phosphoenolpyruvate (PEP) carboxylase, malic enzymes, and PEP carboxykinase, are present in the genomic analysis (14). Enzymes for fatty acid metabolism such as acetyl-CoA carboxlases, malonyl Co-ACP transacylase, 3-ketoacyl ACP-synthase, and 3-ketoacyl ACP-reductase are present (4). Enzymes involved in PHA synthesis like β-ketoacyl-CoA thiolase and acetoacetyl-CoA dehydrogenase were also identified in the genome sequence (6). Therefore, this strain can be applied to sequester CO_2_ and value-added products.

### Accession number(s).

This whole-genome shotgun project has been deposited at DDBJ/ENA/GenBank under the accession no. MBDW00000000. The version described in this paper is version MBDW01000000.
